# Editorial: Ageing and physical activity: a multidimensional approach with new technology

**DOI:** 10.3389/fspor.2025.1549638

**Published:** 2025-02-13

**Authors:** D. Cerasola, G. Giglia, M. Bellafiore

**Affiliations:** ^1^ Department of Psychology, Educational Science and Human Movement, University of Palermo, Palermo, Italy; ^2^ Department of Biomedicine, Neuroscience and Advanced Diagnostics, Section of Human Physiology, University of Palermo, Palermo, Italy

**Keywords:** exergame, virtual reality, ageing, physical activity, exercise

**Editorial on the Research Topic**
Ageing and physical activity: a multidimensional approach with new technology

In high-income countries, people are getting older and, according to recent studies, by 2030 people aged 65–85 will reach 70 million (1).

Ageing is a natural process characterized by the progressive degeneration physiological parameters and related to a decline in functional capacity to perform basic and advanced activities. Because the principal ageing risk factor is the sedentary lifestyle, an active lifestyle is considered as an import factor to maintain health status and prevent the onset of disease.

Physical exercise in the elderly produces positive effects: reduces the risk of developing many chronic conditions, cardiovascular, metabolic, and tumoral diseases and reduces the development risk of hypertension, and osteoporosis (Corrao et al.).

The COVID-19 pandemic imposed radical changes in lifestyle with decrease in physical exercise. However, the COVID-19 period, also give us new strategies to maintain an active lifestyle with a strong development in new tecnologies in smartphone and videogames (exergames) (2). The special issue topic aims to identify studies investigating new tecnologies to improve the correct lifestyle and contrast the negative effect of ageing.

Ramalho et al. propose a reflection on the impact of virtual reality (VR) on our aging population with particular refer to relationship between VR and physical exercise. The authors underline how VR brings opportunities and challenges in elderly. Indeed, virtual reality can have positive psychological implications such as the ability to awaken memories and emotions and lead through virtual games to reduce sedentary lifestyle. However, in the elderly, potential concerns may arise of emotional disconnections or distorted perceptions of reality. Therefore, the correct use of VR by the elderly and its effectiveness will depend on the ability to manage the relationship between technological innovation and human emotions.

In his study, Lee-Confer focuses on the relationship between arms movements and balance to fall prevention. Specifically, Lee-Confer aims to understand how physical therapy protocols in target arm movements may be more effective at preventing falls than exercises that target the legs.

The results of the study show that during falls, the lower limbs do not produce lateral movement. In contrast, the arms have large degrees of movement and their abduction movement reduces falls by 200%+ during a slide. However, in older adults, abduction movement is reduced by 37.5% compared to young adults. Thus, this study proposes to combine leg training with high-speed and ballistic training to improve the proportion and size of type II deltoid fibers. This would improve the ability of older adults to prevent falls.

The study proposed by Navarro et al. aimed to investigate the acceptance of the use of a mobile robot (MTR) used to physical activity control in isolated older by students and teachers of physical activity adapted (APA). A questionnaire measuring different psychological variables, based on the technology acceptance model (TAM), was proposed to 334 participants. The results showed that both students and teachers did not have significant differences for the acceptance of MRT with the items on ease of use, perceived pleasure and intention to use the MTR lower than the mean of the scale. while the perceived usefulness for the elderly was higher than the mean of the scale. While, Navarro et al. propose to deepen the trend of acceptance of APA teachers after the effective use of the MTR.

In the fourth study, Ciaccioni et al. investigate older adults training judo. Specifically Ciaccioni et al. show the relationship between perceived knowledge and training needs. This study had 470 international judo coaches participating in an online questionnaire with 35 different items (aging process; safety and first aid; organization and environment; physiology and fitness; psychology and mental health; teaching and training). The results showed the crucial role of coaches' knowledge and educational needs in facilitating effective and safe judo training programs for older adults. Furthermore coaches with higher judo expertise, extensive experience, higher levels of education and involvement with older practitioners showed greater general knowledge, underlining the value of expertise and practical experience. In addition the study indicates how they should be developed on the design of targeted educational interventions and on the investigation of the impact of specialized training instruction on the quality of judo programs for older people.

In the last study, Palumbo et al. investigated the reduction of the decline of functional physiological parameters related to aging by relating health-related parameters in competitive (C) and physically active (A) elderly people who performed the same amount of weekly physical activity (PA). 34 people took part in the study, divided into 17 competitive and 17 active and matched by age: 8 participants younger than 70 years and 9 older than 70 years for both groups and amount of PA. Body composition, maximum leg and arm strength, balance, reaction time, efficiency of leg and arm exercise, estimated VO2max, VO2/HR relationships, perception of quality of life and sleep were measured.

The results show that group C dedicated more time to intense physical activity compared to A, also showing a better body composition, greater maximum leg strength and a tendency to increase arm strength and a better estimated VO2max. Similar results were shown for the parameters of reaction time, efficiency of leg and arm cycling, balance, perception of quality of life and sleep.

In conclusion, older adults engaged in competitive training show more advantages than those who lead active lives highlighting the importance of adopting active lifestyles to maintain long-term health, high levels of perception of quality of life and reduce age-related decline.

These studies, when seen together, add to the body of knowledge which advocates for a more active lifestyle for our aging populations. They also suggest the need for developers of devices typically associated associated with younger generation to recognize a potential market for their products within the senior population.
